# Cardiac Troponin Is a Predictor of Septic Shock Mortality in Cancer Patients in an Emergency Department: A Retrospective Cohort Study

**DOI:** 10.1371/journal.pone.0153492

**Published:** 2016-04-14

**Authors:** Zhi Yang, Aiham Qdaisat, Zhihuang Hu, Elizabeth A. Wagar, Cielito Reyes-Gibby, Qing H. Meng, Sai-Ching J. Yeung

**Affiliations:** 1 Department of Emergency Medicine, The University of Texas MD Anderson Cancer Center, 1515 Holcombe Blvd., Houston, Texas 77030, United States of America; 2 Department of Intensive Care, Guangzhou First People’s Hospital, Guangzhou Medical University, 1 Panfu Road, Guangdong, People’s Republic of China; 3 Department of Medical Oncology, Sun Yat-sen University Cancer Center, 651 Dongfeng East Rd., West F16, Guangzhou, Guangdong 510060, People’s Republic of China; 4 Laboratory Medicine, The University of Texas MD Anderson Cancer Center, 1515 Holcombe Blvd., Houston, Texas 77030, United States of America; 5 Department of Endocrine Neoplasia and Hormonal Disorders, The University of Texas MD Anderson Cancer Center, 1515 Holcombe Blvd., Houston, Texas 77030, United States of America; Juntendo University Nerima Hospital, JAPAN

## Abstract

**Background:**

Septic shock may be associated with myocardial damage; however, the prognostic value of cardiac enzymes in cancer patients with septic shock is unknown. In this study, we evaluated the prognostic significance of cardiac enzymes in combination with established prognostic factors in predicting the 7-day mortality rate of patients with septic shock, and we constructed a new scoring system, Septic Oncologic Patients in Emergency Department (SOPED), which includes cardiac enzymes, to predict 7-day mortality rates.

**Methods and Findings:**

We performed a retrospective cohort study of 375 adult cancer patients with septic shock who visited the emergency department of a comprehensive cancer center between 01/01/2004 and 12/31/2013. The 7-day and 28-day mortality rates were 19.7% and 37.6%, respectively. The creatine kinase myocardial band fraction and troponin-I were significantly higher in patients who died in ≤7 days and ≤28 days than in those who did not. In Cox regression models, troponin-I >0.05 ng/mL plus Predisposition, Infection, Response, and Organ Failure (PIRO2011) or Mortality in Emergency Department Sepsis (MEDS) score was a significant predictor of survival for ≤7 days. With our new SOPED scoring system, the receiver operating characteristic area under the curve was 0.836, higher than those for PIRO2011 and MEDS.

**Conclusions:**

Troponin-I >0.05 ng/mL was an important predictor of short-term mortality (≤7 days). The SOPED scoring system, which incorporated troponin-I, was more prognostically accurate than were other scores for 7-day mortality. Large multicenter studies are needed to verify our results and prospectively validate the prognostic performance of the SOPED score.

## Introduction

Sepsis is a systemic inflammatory response syndrome that is triggered by a variety of infectious and non-infectious conditions [1]. Extensive research on sepsis has resulted in a promising management strategy, namely “early goal-directed therapy” [2]. Nonetheless, managing sepsis remains a clinical challenge. The Surviving Sepsis Campaign and subsequent research have produced guidelines [3, 4] that clearly define the importance of the “golden hour”—that is, treatment within 1 hour of the recognition of septic shock—in the clinical management of sepsis. More than 50% of hospital admissions occur through the emergency department (ED); thus, the “golden hour” occurs in the ED in most cases. The mean mortality rate for hospitalized sepsis patients admitted through the ED ranges from 5.5%–12.5% [5–7]; therefore, early recognition of sepsis and septic shock may dramatically improve survival outcomes [8]. An efficient evaluation system is particularly needed for patients with cancer, who are especially at risk for developing sepsis because of possible alterations in immune and inflammation response caused by cancer and its treatment.

Currently, numerous scoring systems are used to predict mortality among critically ill adults, from the time-tested Acute Physiology and Chronic Health Evaluation (APACHE) scoring system, first developed in 1981 [9], to the recent Sepsis Severity Score [10]. Most of these systems derive their data from intensive care unit (ICU) patient records and use the worst value for each physiologic parameter in the past 24 hours; unfortunately, this timeframe is unavailable to ED physicians. The only 2 scoring systems that use data that are readily available in the ED are the Mortality in Emergency Department Sepsis (MEDS) score, which was developed in 2003 [5], and the 2011 revised Predisposition, Infection, Response, and Organ Failure (PIRO2011) system [11]. However, all of these scoring systems focus on the 28-day or in-hospital mortality rates of patients with sepsis; none of them address short-term (≤7 days) mortality.

Myocardial dysfunction is an important feature of severe sepsis. Troponin, a protein that consists of 3 subunits (troponin-I [TnI], troponin-C [TnC], and troponin-T [TnT]), is essential to the contractile mechanism of cardiac and skeletal muscles. Cardiac troponins can be elevated in sepsis patients [12–14]. In septic shock, serum TnI concentration has been correlated with myocardial dysfunction [15, 16] and increased mortality [13]. Other studies have confirmed the relationship between troponins and clinical outcomes for patients with sepsis [16, 17]. Two recent meta-analyses confirmed that cardiac troponin levels during sepsis are prognostic of mortality [18, 19]. Brain natriuretic peptide (BNP) is another biomarker of myocardial function, and a meta-analysis concluded that an elevation in BNP or N-terminal pro-B-type natriuretic peptide is associated with increased mortality in sepsis patients [20]. Despite these associations, none of the scoring systems include the biomarkers of myocardial damage, such as cardiac troponins, creatine kinase (CK), and the myocardial band (MB) fraction of CK (CK-MB).

Although the prognostic association between increased cardiac troponins and sepsis has been recognized in the general patient population, it is not known whether this association is also valid in patients with cancer. Moreover, it is unknown how an elevation of serum cardiac troponin levels can be used in the ED setting to predict short-term survival in cancer patients with sepsis. Thus, our overall goal was to determine the prognostic significance of cardiac enzymes in a new prognostic scoring system for septic shock. This study aimed to 1) identify the association between cardiac enzymes and short-term outcomes in cancer patients with septic shock; 2) evaluate the prognostic significance of cardiac enzymes in combination with established scoring systems (APACHE II, Sequential Organ Failure Assessment [SOFA], MEDS, PIRO2009, PIRO2011, and PIRO2013) in predicting the 7-day mortality rate of cancer patients with septic shock; and 3) construct a scoring system based on information readily available in the ED (including cardiac enzyme levels) to predict 7-day mortality rates. Here, we report the results from a retrospective analysis of cancer patients with septic shock; we demonstrate the significance of TnI as a prognostic factor and include it in a new prognostic scoring system (Septic Oncologic Patients in an Emergency Department [SOPED]).

## Methods

### Research design and patients

We conducted a retrospective review of patients who visited the ED at The University of Texas MD Anderson Cancer Center (Houston, Texas) between January 1, 2004, and December 31, 2013, under a clinical research protocol (protocol number DR08-0066) approved by the MD Anderson institutional review board. The research was conducted in compliance with Health Insurance Portability and Accountability Act regulations and the Helsinki Declaration (WMA Declaration of Helsinki—Ethical Principles for Medical Research Involving Human Subjects). Written informed consent was not obtained for this retrospective study, and patient records and information were anonymized and de-identified prior to analysis.

Our ED has approximately 22,000 visits per year, and 51% of these visits result in hospital admission. Using the hospital billing database and a diagnosis of septic shock (ICD9-code 785.52, 2014) and without diagnosis of myocardial infarction (ICD9-code 410.00–410.92, 2014), we identified 450 unique patients out of 155,032 ED visits between 1/1/2004 and 12/31/2013 for this retrospective chart review study. The following exclusion criteria were applied: age <18 years, no evidence of infection, absence of shock in the ED, cardiac arrest and CPR in the ED, and lack of full access to the medical records.

We used the 2001 Society of Critical Care Medicine/European Society of Intensive Care Medicine/American College of Chest Physicians/American Thoracic Society/Surgical Infection Society International Sepsis Definitions Conference definitions of sepsis in this study. Sepsis is defined as a systemic inflammatory response syndrome triggered by a variety of infectious and non-infectious conditions. Severe sepsis is defined as sepsis complicated by organ dysfunction, whereas septic shock is severe sepsis complicated by hypotension that is refractory to adequate volume resuscitation in the absence of an alternate cause [1].

### Data collection and definition of endpoints

The following data were collected from patients’ medical records: patient age, source of infection, altered mental status, vital signs (temperature, blood pressure, heart rate, respiratory rate, and oxygen saturation), comorbidities (liver disease, congestive heart failure, chronic obstructive pulmonary disease, and diabetes), drug usage (previous antibiotics and prednisone), and laboratory data (complete blood counts with differential, electrolytes, creatinine, blood urea nitrogen [BUN], liver function tests, cardiac biomarkers [BNP, TnI, CK, and CK-MB], arterial pH, partial pressure of oxygen, and lactate).

The Charlson Comorbidity Index (CCI) [21] was used to control for the contribution of comorbid conditions to mortality. The APACHE II score, SOFA score, MEDS score, PIRO2009 score, PIRO2011 score, and PIRO2013 score were calculated as previously described [5, 11, 21–25]. In multivariate regression models, a binary categorical covariate for comorbidity was used by defining it as low for an age-unadjusted CCI score of ≤ 4 and high for a CCI score > 4.

Unlike many other scoring systems that use the worst values in the first 24 or 48 hours (often after admission to the ICU), we used the worst values obtained while the patient was still in the ED. If a variable or parameter was not available, it was entered as 0 and handled in a way similar to that used in the APACHE scoring system [9].

We used the 7-day mortality rate as the primary outcome to predict the risk of short-term mortality. As MEDS, Sepsis Severity Score, PIRO2009, PIRO2011, PIRO2013, SOFA, and APACHE II scores predict the risk of 28-day mortality or in-hospital mortality, we used the 28-day mortality rate as the secondary endpoint in our research. Therefore, we performed a time-to-event analysis using death within 7 days after ED presentation as the event; patients who remained alive 7 days after ED presentation were censored in this analysis. A similar analysis was performed using the 28-day mortality rate.

### Data analysis

Patient data were summarized using descriptive statistics. Student’s t-test or Wilcoxon signed-rank test was used to compare continuous variables between 2 groups, where appropriate. Logarithmic transformation was applied to variables with skewed distributions. Ratios of distribution among categories were evaluated by the chi-square test or Fisher exact test, where appropriate. Statistical significance for rejection of the null hypothesis was set at *P* < 0.05. A Kaplan–Meier survival analysis with a log-rank test was used to evaluate the significance of cardiac enzymes in predicting 7-day and 28-day mortality. Six scoring systems (APACHE II, SOFA, MEDS, PIRO2009, PIRO2011, and PIRO2013) and 4 cardiac biomarkers (BNP, CK, CK-MB, and TnI) were examined for correlations, and the Pearson's correlation coefficients were reported in a correlation matrix. Similar analyses were conducted in subgroups of patients enrolled between 2009–2013 and 2011–2013 to examine whether advances in sepsis treatment and resuscitation over time during the study had changed the prognostic value of cardiac enzymes.

Cox proportional hazard models were used to determine whether cardiac biomarkers were significantly predictive of 7-day and 28-day mortality rates, independent of different scoring systems. As we built a model of 7-day mortality prediction, we used the random forest machine learning method to identify the parameters that were predictive of 7-day mortality among a list of 35 parameters; these parameters included cardiac biomarkers, laboratory data, and individual parameters used in the calculation of the 6 scores (CK-MB > 6.3 ng/mL, TnI > 0.05 ng/mL, lactate > 4 mmol/L, respiratory rate > 20 breaths/min, potassium > 5.0 mmol/L, potassium < 3.5 mmol/L, BUN > 20 mg/dL, creatinine > 1.3 mg/dL, age > 65 years, pulse > 100 beats per minute [bpm], sodium > 147 mmol/L, platelets < 150,000,000/L, CCI (unadjusted for age) > 4, sex, race, metastatic malignancies, respiratory failure or hypoxemia, hypothermia, pneumonia, diabetes, hyperglycemia, more than 1 infection, altered mental status, any infection [excluding skin or soft tissue infection], use of glucocorticoids, atherosclerosis, bacteremia, previous antibiotics use, chronic liver disease, hospital-acquired infection, fever, type of malignancy, chronic obstructive pulmonary disease, chronic non-malignant hematologic disease, and neutropenia). On the basis of the average importance plots, the top parameters that contributed to prediction accuracy were chosen to construct a new Cox regression model. The significant (*P* < 0.05) predictors were used to construct a new scoring system (SOPED) using the Z coefficients multiplied by 2 and then rounded to whole numbers as points to be added up to a total score. The performance of the SOPED score at predicting 7-day mortality was evaluated by the Hosmer–Lemeshow “goodness-of-fit” test. The receiver-operating characteristic (ROC) was used to compare the prediction accuracy of SOPED with that of the other 6 scoring systems using the DeLong method (pROC R-package).

All statistical analyses were performed using R software (version 3.2.0, The R Foundation, http://www.r-project.org).

## Results

### Patient characteristics

**Fig 1:**
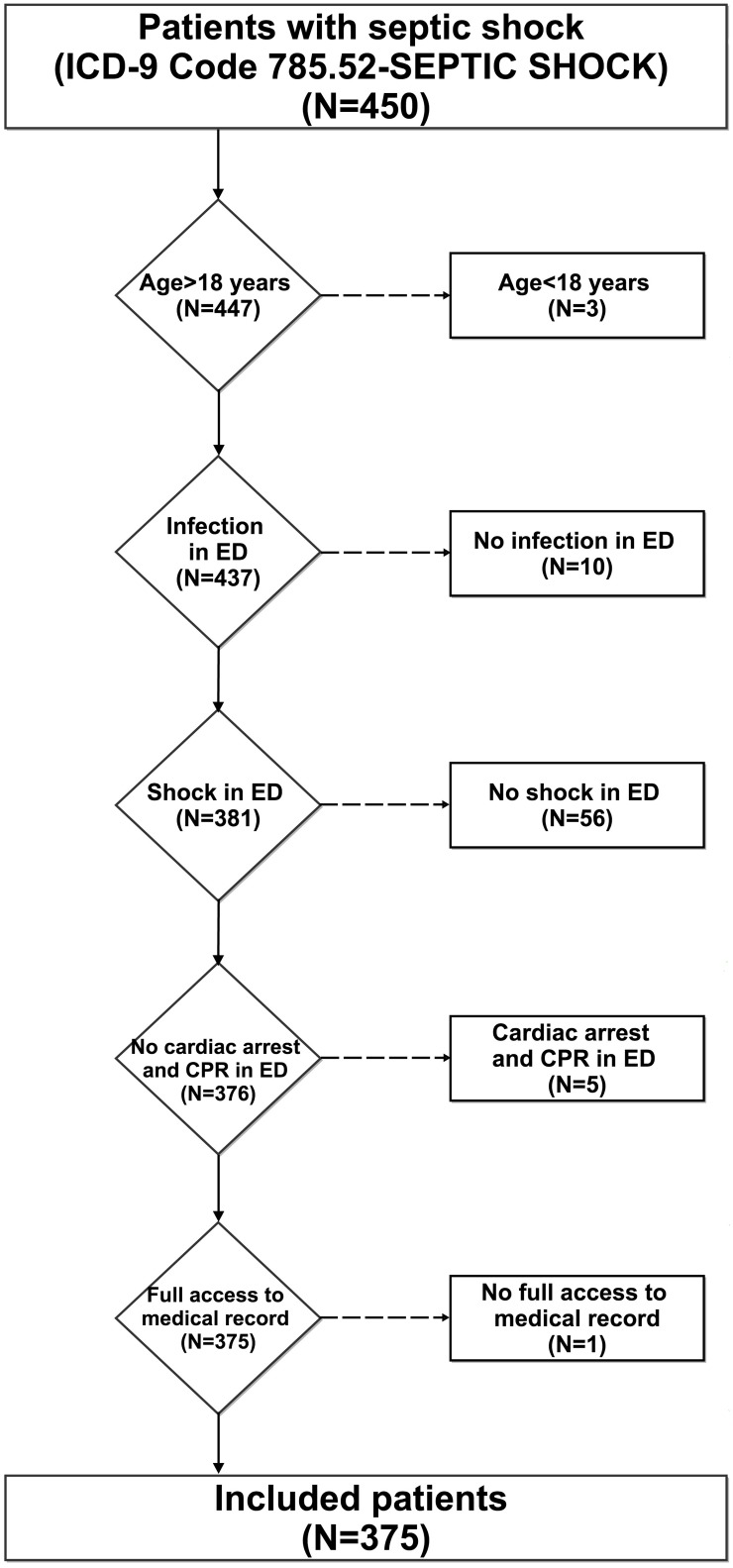
Cases of septic shock in the ED at MD Anderson from 2004 to 2013. The number of cases and the reasons for exclusion from review and analysis are indicated.

During the study period, 1,380 patients were diagnosed with sepsis. Using the hospital billing database and a diagnosis of septic shock, with no diagnosis of myocardial infarction, we identified 450 unique patients. Of these, 75 were excluded from further review and analysis for one of the following reasons: age < 18 years (n = 3), no evidence of infection (n = 10), absence of documented shock in the ED (i.e., no hypotension and lactate < 4 mmol/L (n = 56)), cardiac arrest and cardiopulmonary resuscitation in the ED (n = 5), and lack of full access to the medical records (n = 1). A full review was performed of the final study population of 375 patients (Fig 1).

The 7-day mortality rate was 19.7% (74 of 375 patients). The 28-day mortality rate was 37.6% (141 of 375), with more than half of the deaths occurring within 7 days. The characteristics of the non-survivors and survivors are shown in Table 1. There were no statistically significant (*P* < 0.05) differences between non-survivors and survivors in age, sex, race, percentages of hematologic malignancies and solid cancers, or age-adjusted and age-unadjusted CCI when 7-day mortality and 28-day mortality rates were analyzed.

**Table 1:** Characteristics of the non-survivors and survivors.

Mortality by patient characteristic	Survivors	Non-survivors	*P* value
Age (years) (mean ± SD)			
7-day	56.6 ± 14	59.1 ± 15	0.22
28-day	55.8 ± 14	58.3 ± 15	0.11
Male, % (n/total)			
7-day	60.80 (183/301)	55.41 (41/74)	0.35
28-day	62.39 (146/234)	54.61 (77/141)	0.12
Race, % (n/total)			
White			
7-day	56.81 (171/301)	62.16 (46/74)	0.48
28-day	56.41 (132/234)	58.87 (83/141)	0.60
Black			
7-day	12.96 (39/301)	16.22 (12/74)	0.59
28-day	11.54 (27/234)	15.60 (22/141)	0.25
Solid cancer, % (n/total)			
7-day	51.83 (156/301)	56.76 (42/74)	0.89
28-day	49.57 (116/234)	57.45 (81/141)	0.14
CCI unadjusted for age > 4, % (n/total)			
7-day	49.20 (148/301)	62.16 (46/74)	0.06
28-day	48.72 (114/234)	56.74 (80/141)	0.13
CCI adjusted for age > 6, % (n/total)			
7-day	40.53 (122/301)	45.95 (34/74)	0.47
28-day	39.32 (92/234)	45.39 (64/141)	0.25

#### Cardiac enzyme levels were higher in non-survivors than in survivors

**Fig 2:**
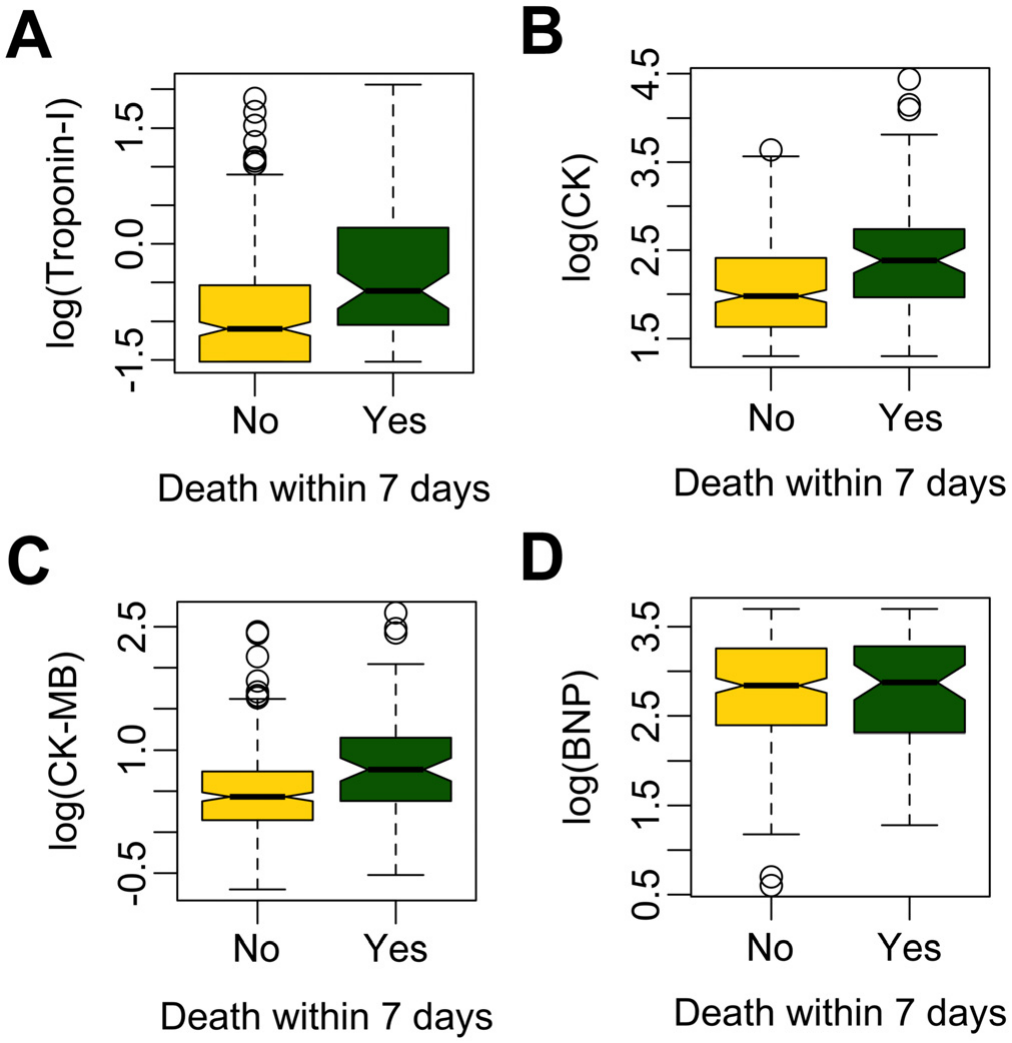
Comparison of cardiac biomarkers between cancer patients with septic shock admitted to the ED who survived for > 7 days and those who died within 7 days. The box plots of the logarithms of troponin-I (A), CK (B), CK-MB (C), and BNP (D) are shown.

A comparison of cardiac biomarkers between non-survivors who died of septic shock within 7 days or 28 days versus those who survived showed that log(TnI) was significantly higher in non-survivors (*P* < 0.001, t test for both; Fig 2A and S1A Fig). Log(CK) was similarly higher in non-survivors than in survivors (*P* < 0.001 for both; Fig 2B and S1B Fig), as was log(CK-MB) (*P* < 0.001 for both; Fig 2C and S1C Fig). Using the non-parametric Wilcoxon test, we found that TnI was significantly (*P* < 0.001) higher in non-survivors (median, 3.29 ng/mL in 7-day mortality; median, 2.34 ng/mL in 28-day mortality) than in survivors (median, 1.21 ng/mL in 7-day mortality; median, 1.18 ng/mL in 28-day mortality). CK was similarly higher in non-survivors (1350.5 U/L for 7-day mortality and 818.6 U/L for 28-day mortality) than in survivors (266.7 U/L for 7-day mortality and 276.8 U/L for 28-day mortality), as was CK-MB (median values of 25.8 ng/mL and 7.7 ng/mL for non-survivors and survivors, respectively, for 7-day mortality, and 17.7 ng/mL and 7.4 ng/mL for non-survivors and survivors, respectively, for 28-day mortality) (all *P* < 0.001). For log(BNP) (Fig 2D and S1D Fig) and BNP, no significant differences were found between the 2 groups.

### Cardiac enzymes were associated with an increased 7-day mortality rate in cancer patients with septic shock

**Fig 3:**
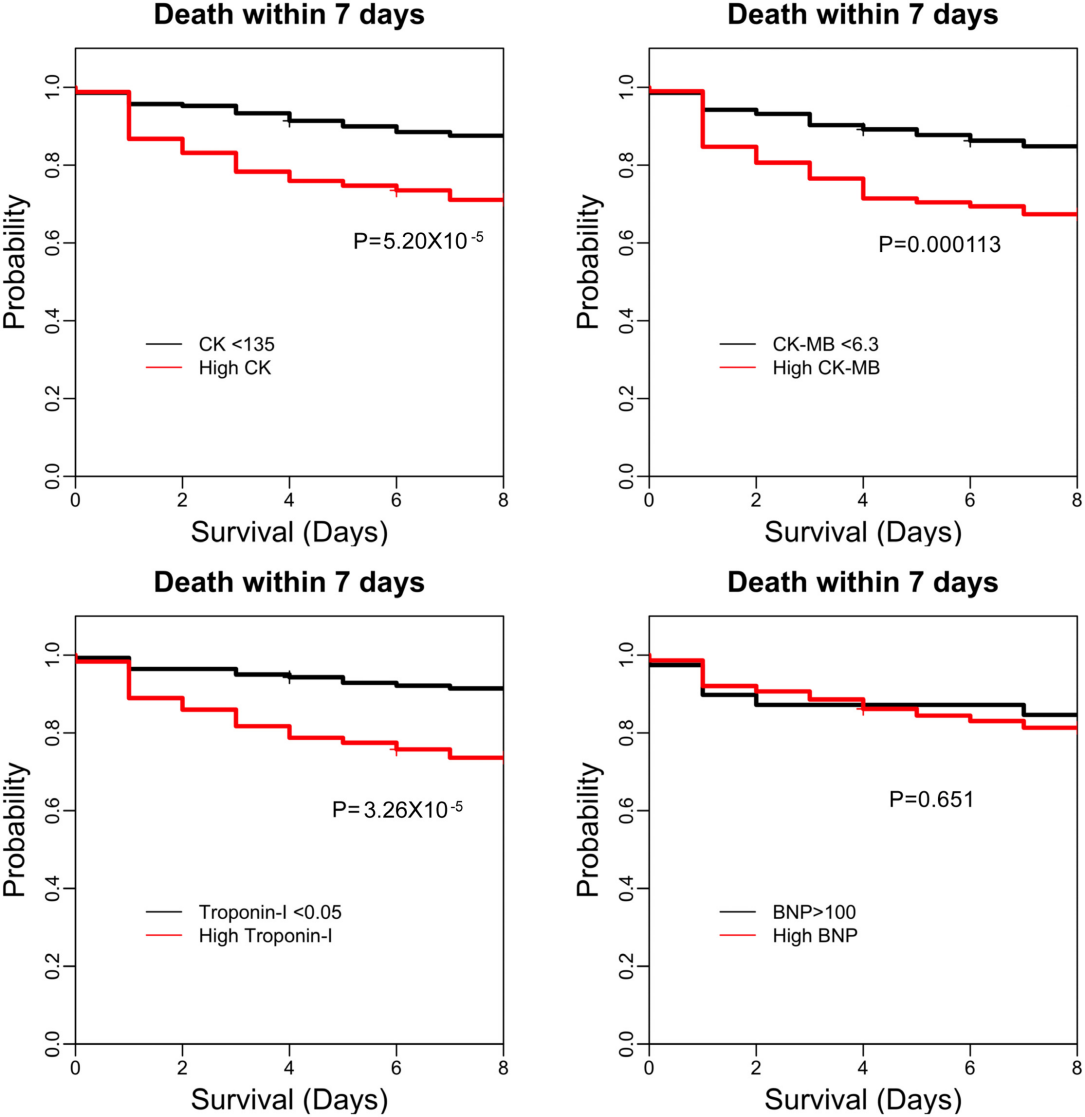
Kaplan-Meier survival analysis of mortality events for the first week after cancer patients were admitted through the ED for septic shock. Patients were divided into groups using cut-off values for categorization, as labeled. Survival curves are shown for the analyses of CK (left upper panel), CK-MB (right upper panel), troponin-I (left lower panel), and BNP (right lower panel).

A Kaplan–Meier survival analysis showed that patients with elevated CK, CK-MB, and TnI levels had a higher risk for mortality than did patients with normal levels, both for 7-day mortality and 28-day mortality (all *P* < 0.001) (Fig 3 and S2 Fig). There was no significant (*P* = 0.651) difference in the risk of mortality between patients with abnormal or normal BNP (Fig 3 and S2 Fig, right lower panel). Similar analyses of the subgroups of patients between 2009–2013 and 2011–2013 confirmed this finding (S1 Table). Therefore, advances in sepsis treatment and resuscitation did not appear to have changed the prognostic value of cardiac enzymes between the early and later parts of the study period.

### Correlations between sepsis prognostic scores and cardiac biomarkers

We used a correlation matrix to tabulate the pairwise correlations among sepsis prognostic scores (APACHE II, SOFA, MEDS, PIRO2009, PIRO2011, and PIRO2013) and logarithms of cardiac biomarkers (log(BNP), log(CK), log(CK-MB), and log(TNI)) (S3 Fig). APACHE II, SOFA, MEDS, PIRO2009, and PIRO2011 had moderate positive correlations, with correlation coefficients ranging from 0.44 to 0.66. PIRO2013 did not correlate well with the other scores. As expected, the 2 prognostic scores developed from ED patients (MEDS and PIRO2011) correlated well with each other. Among the cardiac biomarkers, strong correlations existed between log(CK) and log(CK-MB) and between log(CK-MB) and log(TnI). The correlations between cardiac enzymes and sepsis prognostic scores were weak (correlation coefficients close to 0.30). Cardiac enzymes were associated with increased mortality in cancer patients with septic shock and were weakly correlated with sepsis prognostic scores; thus, we next determined whether cardiac enzymes were independent prognostic predictors of 7-day mortality rates.

### Cox regression analysis of 7-day mortality rates of cancer patients with septic shock admitted through the ED

We determined whether TnI or CK-MB was an independent predictor of 7-day mortality in the presence of PIRO2011 or MEDS while controlling for age (older than 65 years vs. 65 years or younger), race (black vs. non-black), sex (male vs. female), type of malignancy (hematologic malignancies vs. solid tumors), and comorbidity (age-unadjusted CCI > 4 vs. ≤ 4). For the Cox model that included PIRO2011 and TnI, TnI was a significant (*P* = 0.034) independent predictor of 7-day mortality (odds ratio, 2.05; 95% confidence interval [CI], 1.06–3.97) (S2 Table). For the Cox model that included PIRO2011 and CK-MB, CK-MB was a near-significant (*P* = 0.055) predictor (S3 Table). For the model that included MEDS and TnI, TnI was also significant (*P* < 0.001; odds ratio, 3.87; 95% CI, 2.01–7.46) (S4 Table), and for the model that included MEDS and CK-MB, CK-MB was significant (*P* = 0.001; odds ratio, 2.21; 95% CI, 1.37–3.57) (S5 Table). Therefore, we continued to evaluate log(TnI) and log(CK-MB) as independent prognostic predictors.

### Factors that are predictive of 7-day mortality

We used the random forest method to analyze the relative importance of factors used to determine sepsis prognostic scores in predicting 7-day mortality rates. According to the average importance plots, the mean decreases in accuracy suggested that lactate > 4 mmol/L, creatinine > 1.3 mmol/L, respiratory rate > 20 bpm, potassium > 5 mmol/L, metastatic malignancy, pneumonia, TnI > 0.05 ng/mL, age > 65 years, BUN > 20 mg/dL, respiratory failure/hypoxemia, and hypothermia were the top 11 factors (S4 Fig). We then constructed a Cox regression model using these factors to predict 7-day mortality rates; 8 factors (lactate > 4 mmol/L, creatinine > 1.3 mmol/L, respiratory rate > 20 bpm, potassium > 5 mmol/L, metastatic malignancies, pneumonia, TnI > 0.05 ng/mL, and age > 65 years) were significant (*P* < 0.05) (data not shown).

### Construction of a new scoring system

**Fig 4:**
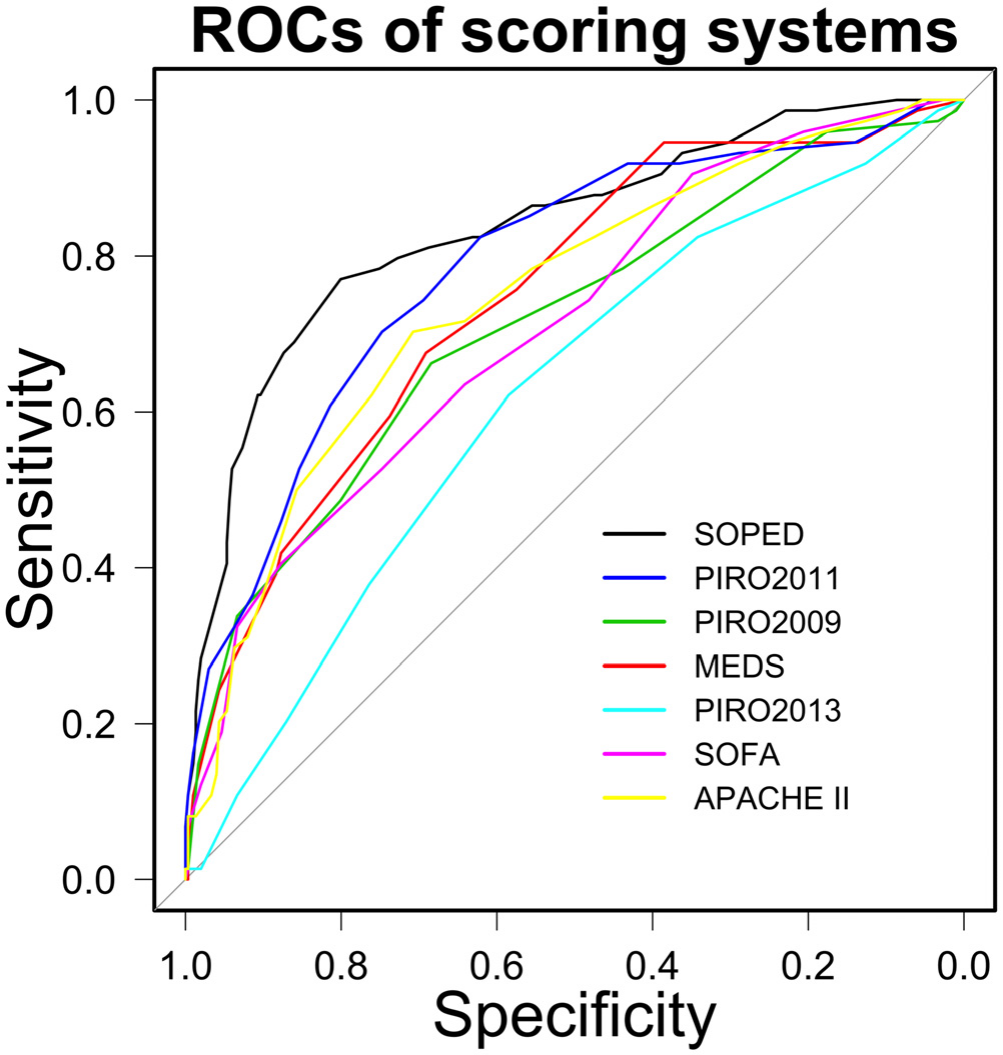
Comparison of the ROC analysis between the SOPED score and other sepsis prognostic scores applied to cancer patients with septic shock admitted through the ED. The curves are colored as labeled.

The status of these 8 factors, as well as the age-unadjusted CCI, can readily be determined in the ED. To determine the weighing multiplier for each factor in a new prognostic score, we used 2 times the Z coefficients (Table 2), rounded to the nearest integer. We used these factors to create a new instrument, the Septic Oncologic Patients in Emergency Department (SOPED) score, to predict the 7-day mortality rate of cancer patients in the ED with septic shock. The *P* value of the Hosmer–Lemeshow “goodness-of-fit” test was 0.58 and the delta value of the K-fold cross-validation was 0.122. The model passed these tests. An ROC analysis of SOPED for 7-day mortality showed an area under the curve (AUC) of 0.84, with a 95% CI of 0.78–0.89. According to the turning points of the AUC, 3 risk groups can be categorized: low (total SOPED points, 0–19; 7-day mortality rate, 5.6%), medium (total SOPED points, 20–30; mortality rate, 14.5%), and high (total SOPED points, 31–47; mortality rate, 62.2%). The predictive performance of SOPED for 7-day mortality was compared with that of other sepsis prognostic scores by ROC analysis (Fig 4). The AUC for SOPED was the highest (DeLong test, *P* < 0.05 for all pairwise comparisons).

**Table 2:** Multivariate Cox regression model and proposed SOPED score.

Patient characteristic	Z	95% CI	*P* value	SOPED Points
Age > 65 years	2	1.00–1.04	0.047	4
Respiratory rate > 20 bpm	3.6	1.13–1.61	0.001	7
Potassium > 5 mmol/L	2.4	1.15–3.32	0.013	5
Creatinine > 1.3 mg/dL	3.6	1.50–4.52	< 0.001	7
Troponin-I > 0.05 ng/mL	1.9	1.02–3.83	0.043	4
Lactate > 4 mmol/L	4.5	1.23–1.71	< 0.001	9
Pneumonia	1.8	1.02–3.08	0.044	4
Malignancy: distant metastasis	2.4	1.60–117.02	0.017	5
Charlson Comorbidity Index (unadjusted for age) > 4	1.1	0.81–2.14	0.272	2
SOPED Score Total				47

## Discussion

Our analysis of cancer patients with septic shock in the ED showed that myocardial injury is an independent predictor of 7-day and 28-day mortality rates and that TnI was the most robust cardiac biomarker that could be used, in conjunction with a list of other predictive factors that are readily available in the ED, to calculate a new score (SOPED) to predict the 7-day mortality rate. Previous studies [13, 15–17] have demonstrated that myocardial injury is predictive of in-hospital or 28-day mortality in patients with severe sepsis or septic shock. A meta-analysis [20] also showed the significance of cardiac biomarkers in sepsis prognosis. However, no studies have incorporated cardiac enzymes into a prognostic scoring system that is suitable for use in the ED, and no prior studies have been performed in patients with cancer, in whom the immune and inflammation responses may have been altered by the malignancy or treatment. For instance, neutropenia is often present in leukemia patients, often due to chemotherapy. Plasma C-reactive protein [26], interleukin-6 [27], and interleukin-8 [27] concentrations are higher in sepsis patients with neutropenia than in those without.

Our data confirmed that cardiac enzymes are predictive of short-term mortality in cancer patients with septic shock, although BNP was not predictive in our data set. Since BNP can be increased by aggressive intravenous fluid resuscitation or cardiac “pump” dysfunction, one possible explanation for that lack of association of mortality with BNP is that BNP was measured at presentation in the ED (before intravenous fluid resuscitation) in our study but later in the clinical course (in the ICU) in most other studies. Knowing that myocardial injury is an important predictor of sepsis mortality, the next step is to determine how to use biomarkers of myocardial injury to predict sepsis mortality. This is a major knowledge gap, as no studies have incorporated cardiac biomarkers into prognostic tools. No current sepsis prognostic scoring system considers myocardial injury or cardiac biomarkers.

In our investigation, we evaluated APACHE II, SOFA, MEDS, PIRO2009, PIRO2011, and PIRO2013 for the following reasons: 1) APACHE II and SOFA are widely used, classic scoring systems that have been in clinical use for almost 30 years; 2) the PIRO scoring system is a more sophisticated model system that first emerged in 2000 [28] and has repeatedly been modified; and 3) MEDS is one of only 2 systems (the other being PIRO2011) that is based on ED patient data. Although studies have shown the prognostic value of MEDS in patients with sepsis in general [6, 29, 30] and in those with special infections in the ED [31], it performed poorly in several studies that focused on patients with severe sepsis or septic shock [32–34]. Some researchers have tried to improve the prognostic accuracy of MEDS by combining it with sepsis biomarkers such as C-reactive protein, procalcitonin, and interleukin-6, with encouraging results [6, 35, 36]. As for the PIRO scoring systems, only 2 studies in patients with severe sepsis or septic shock used PIRO2009 [37, 38]. PIRO2009 was more accurate than SOFA and was similar in performance to MEDS and APACHE II in ED patients with severe sepsis and septic shock. More work is needed to clarify the importance of PIRO2011, which is derived from ED patients, in high-risk patients with severe sepsis or septic shock.

Although most studies have used 28-day mortality rates as an endpoint, our analysis showed that more than 50% of cancer patients with septic shock who died within 28 days had died within 7 days, and that more than 65% of cancer patients with septic shock who died within 7 days had died within 3 days (50 of 74). As time passes, patients with septic shock may die from other factors that could not have been predicted in the ED. For ED and ICU physicians who need to focus their efforts on sepsis patients with the highest risk for short-term mortality, a prognostic tool for 7-day mortality is more relevant than is a tool for 28-day mortality.

Our study demonstrated that PIRO2011 had the best performance among the 6 scoring systems in predicting 7-day mortality. Because TnI is an important independent predictor of 7-day mortality, the combination of TnI and PIRO2011 significantly improved the predictive ability of PIRO2011. However, PIRO2011 contains 14 factors, indicating that the score is difficult to obtain for ED physicians who have limited time to manage the emergency. In addition, some single factors may dominate in predictions of adverse outcomes. For example, the AUC of the ROC curve for lactate was not significantly different from that for MEDS, APACHE II, and SOFA for the 28-day mortality rate [39]. Therefore, we constructed a user-friendly prognostic scoring system (SOPED) to estimate 7-day mortality rates for cancer patients in the ED with septic shock. SOPED includes TnI, based on results from the random forest method to identify important predictive factors. The performance of this new SOPED score was better than that of the other 6 scoring systems, as shown by an ROC analysis.

Our study had several limitations. This was a single-center study with a small sample size. The results should be verified in a large well-designed multicenter clinical study. During the period of time in which our patients were treated (January 1, 2004, to December 31, 2013), important advances in the management of sepsis occurred [2–4]. Although the overall survival rate of sepsis has improved over the years, elevation of cardiac enzymes remained a significant predictor of 7-day survival and 28-day survival in these patients with septic shock, even in the last 3–5 years of data (S1 Table). The SOPED scoring system was tested in patients with cancer. In future studies, we may also modify the SOPED score to apply to non-cancer patients with septic shock in the ED. We note, however, that the prognosis of cancer patients with septic shock is probably not generalizable to non-cancer patients, because metastasis is a significant predictor of mortality in septic cancer patients, and this factor is clearly absent in patients without cancer. Finally, like most retrospective studies, our study did not provide any mechanistic or experimental data to explain the statistical association between TnI and septic shock mortality.

## Conclusions

Cancer patients in the ED with septic shock who died within 7 days and 28 days had higher cardiac enzyme levels than did patients who survived. Cardiac enzyme levels were independent predictors of mortality within 7 days and 28 days in this patient population. The addition of TnI improved the performance of PIRO2011 in predicting the 7-day mortality rate in these patients. A new SOPED scoring system that incorporated TnI had better prognostic performance than did many other prognostic scores for the 7-day mortality rate. Validation of the SOPED score in future studies is warranted.

## Supporting Information

S1 Fig Comparison of cardiac biomarkers between cancer patients with septic shock admitted through the ED who survived for > 28 days and those who died within 28 days. The box plots of the logarithms of troponin-I (A), CK (B), CK-MB (C), and BNP (D) are shown.
(TIF)

S2 Fig Kaplan-Meier survival analysis of mortality events for the 28 days after cancer patients were admitted through the ED for septic shock. Patients were divided into groups using cut-off values for categorization, as labeled. The survival curves are shown for the analyses of CK (left upper panel), CK-MB (right upper panel), troponin-I (left lower panel), and BNP (right lower panel).
(TIF)

S3 Fig Pairwise correlations of sepsis prognostic scores and logarithms of cardiac biomarkers in the correlation matrix analysis. A correlation matrix is shown. The diagonal panels show the frequency distribution of the respective quantitative values. Panels below the diagonal show scatter plots of the respective values with red lines that follow the scattering. Panels above the diagonal show correlation coefficients. Red asterisks: *P = 0.05–0.01, **P = 0.01–0.001; ***P < 0.001.
(TIF)

S4 Fig Random forest analysis of the relative importance of factors in predicting 7-day mortality in cancer patients in the ED with septic shock. The top 30 factors for the mean decrease in accuracy (left panel) and mean decrease in Gini (right panel) are shown.
(TIF)

S1 Table Log-rank test *P* values for 2009–2013 and 2011–2013 in Kaplan–Meier analyses performed in the same manner as in Fig 3 and S2 Fig. (DOCX)

S2 Table Cox regression model, including PIRO2011 and TnI, for 7-day mortality rate. (DOCX)

S3 Table Cox regression model including PIRO2011 and CK-MB for 7-day mortality rate. (DOCX)

S4 Table Cox regression model including MEDS and TnI for 7-day mortality rate. (DOCX)

S5 Table Cox regression model including MEDS and CK-MB for 7-day mortality rate. (DOCX)

## Abbreviations

APACHE: Acute Physiology and Chronic Health Evaluation score
BNP: brain natriuretic peptide
BUN: blood urea nitrogen
CCI: Charlson Comorbidity Index
CK: creatine kinase
CK-MB: myocardial band fraction of CK
ED: emergency department
ICU: intensive care unit
MEDS: Mortality in Emergency Department Sepsis score
PIRO: Predisposition, Infection, Response, and Organ Failure score
PIRO2009: Predisposition, Infection, Response, and Organ Failure 2009 score
PIRO2011: Predisposition, Infection, Response, and Organ Failure 2011 score
PIRO2013: Predisposition, Infection, Response, and Organ Failure 2013 score
SOFA: Sequential Organ Failure Assessment
SOPED: Septic Oncologic Patients in an Emergency Department score
TnI: troponin-I
TnC: troponin-C
TnT: troponin-T
