# Supplementary figures and images for: Cardiac Troponin Is a Predictor of Septic Shock Mortality in Cancer Patients in an Emergency Department: A Retrospective Cohort Study
Source: PLoS One. 2016 Apr 14;11(4):e0153492. doi: 10.1371/journal.pone.0153492 (PMC4831781; doi:10.1371/journal.pone.0153492)

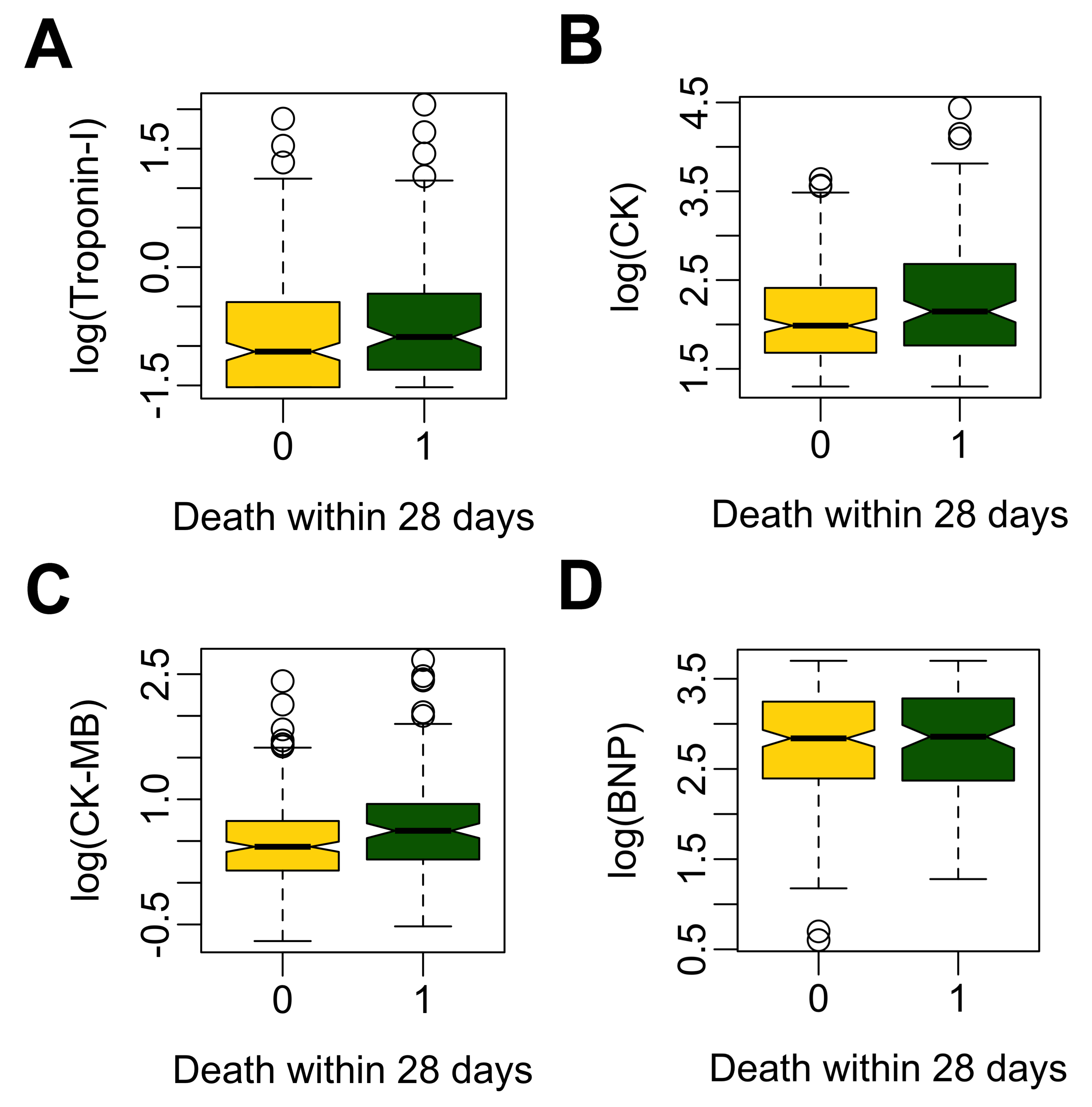

Supplement: S1 Fig — The box plots of the logarithms of troponin-I (A), CK (B), CK-MB (C), and BNP (D) are shown. (TIF) [file pone.0153492.s001.tif]

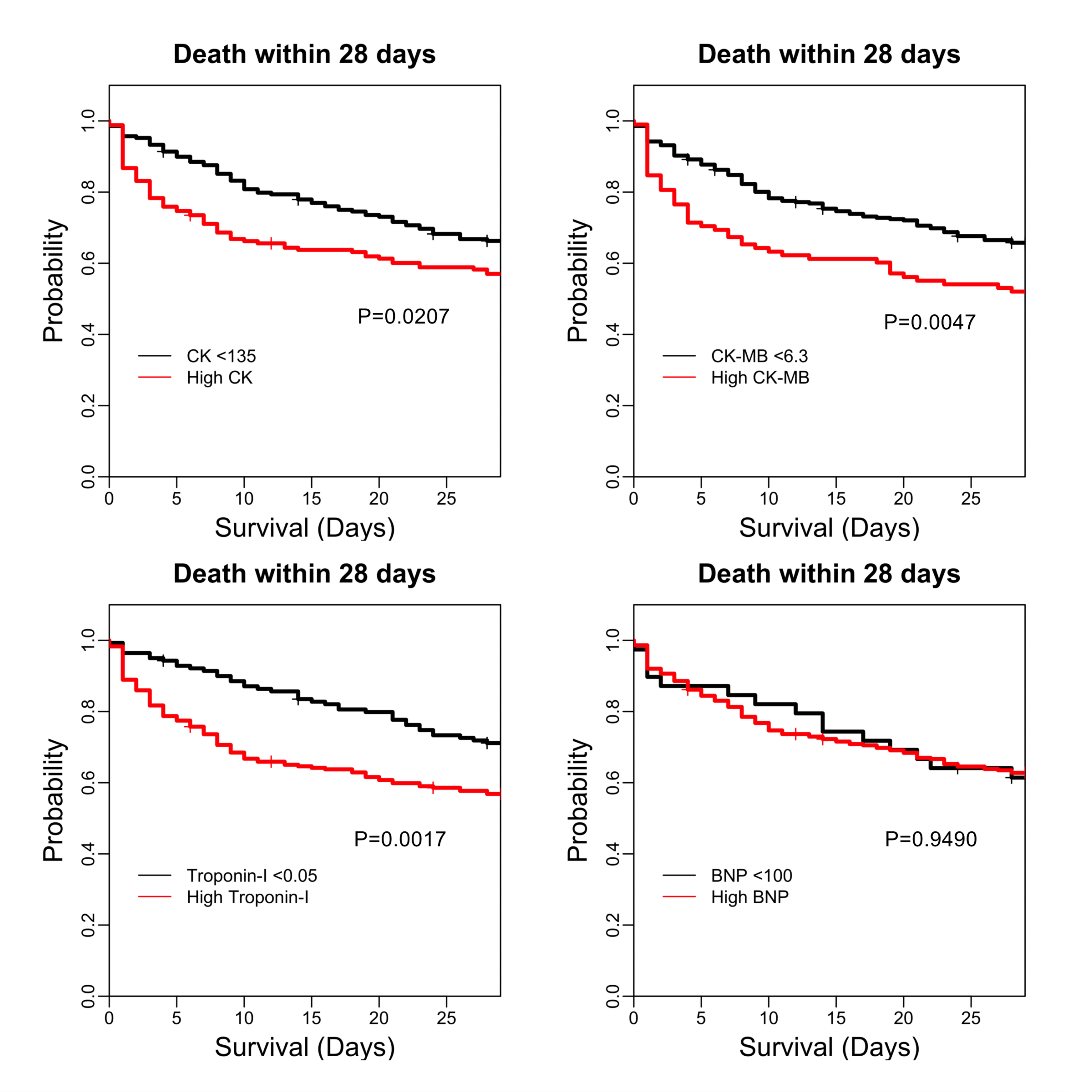

Supplement: S2 Fig — Patients were divided into groups using cut-off values for categorization, as labeled. The survival curves are shown for the analyses of CK (left upper panel), CK-MB (right upper panel), troponin-I (left lower panel), and BNP (right lower panel). (TIF) [file pone.0153492.s002.tif]

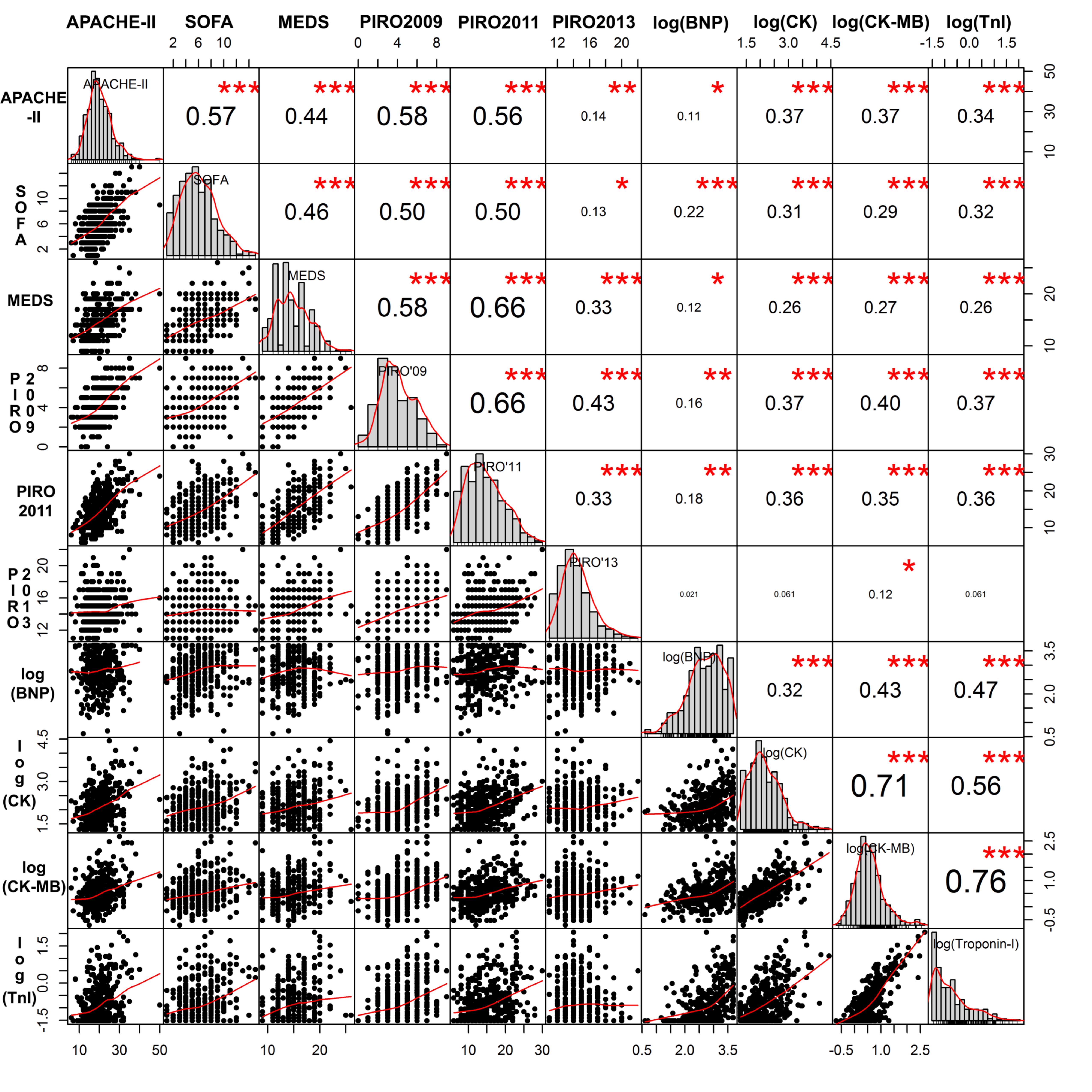

Supplement: S3 Fig — A correlation matrix is shown. The diagonal panels show the frequency distribution of the respective quantitative values. Panels below the diagonal show scatter plots of the respective values with red lines that follow the scattering. Panels above the diagonal show correlation coefficients. Red asterisks: *P = 0.05–0.01, **P = 0.01–0.001; ***P < 0.001. (TIF) [file pone.0153492.s003.tif]

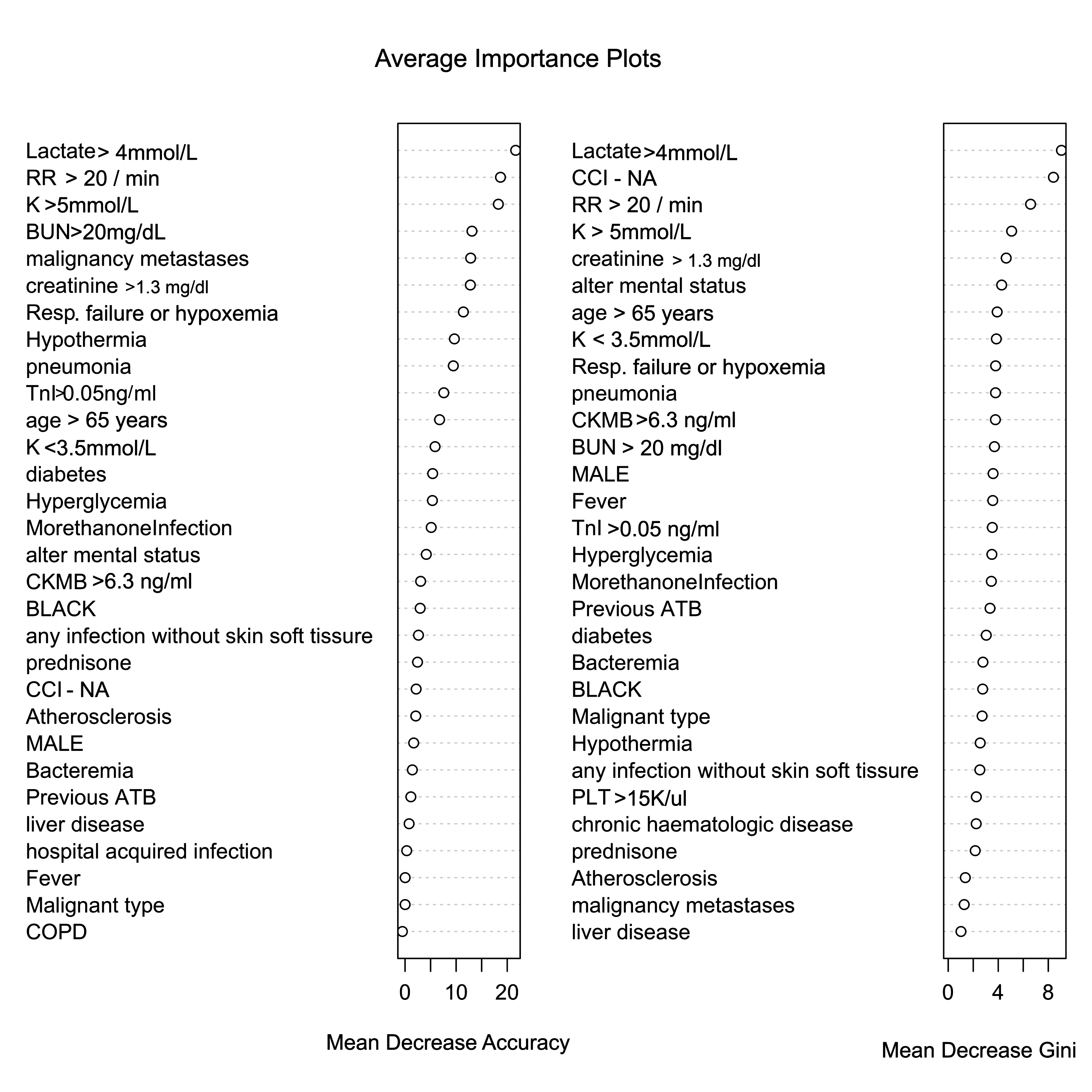

Supplement: S4 Fig — The top 30 factors for the mean decrease in accuracy (left panel) and mean decrease in Gini (right panel) are shown. (TIF) [file pone.0153492.s004.tif]
