# Supplementary material for: Cardiac Troponin Is a Predictor of Septic Shock Mortality in Cancer Patients in an Emergency Department: A Retrospective Cohort Study
Source: PLoS One. 2016 Apr 14;11(4):e0153492. doi: 10.1371/journal.pone.0153492 (PMC4831781; doi:10.1371/journal.pone.0153492)
Supplement: S1 Table — (DOCX) [file pone.0153492.s005.docx]

| **Outcome by factor** | ***P* value** | |
| --- | --- | --- |
|  | **Cases from 2009-2013** | **Cases from 2011-2013** |
| 7-day mortality |  |  |
| CK > 135 U/L | < 0.001 | < 0.001 |
| CK-MB > 6.3 ng/mL | < 0.001 | < 0.001 |
| Troponin-I > 0.05 ng/mL | < 0.001 | < 0.001 |
| BNP > 200 ng/L | 0.857 | 0.781 |
| 28-day mortality |  |  |
| CK > 135 U/L | 0.215 | 0.586 |
| CK-MB > 6.3 ng/mL | 0.040 | 0.050 |
| Troponin-I > 0.05 ng/mL | 0.003 | 0.007 |
| BNP > 200 ng/L | 0.904 | 0.866 |
