# Supplementary material for: Cardiac Troponin Is a Predictor of Septic Shock Mortality in Cancer Patients in an Emergency Department: A Retrospective Cohort Study
Source: PLoS One. 2016 Apr 14;11(4):e0153492. doi: 10.1371/journal.pone.0153492 (PMC4831781; doi:10.1371/journal.pone.0153492)
Supplement: S2 Table — (DOCX) [file pone.0153492.s006.docx]

| **Patient characteristic** | **Odds ratio** | **95% CI** | ***P* value** |
| --- | --- | --- | --- |
| PIRO2011 | 1.22 | 1.16–1.29 | < 0.001 |
| Age > 65 years | 1.01 | 0.99–1.03 | 0.217 |
| Black race | 0.82 | 0.43–1.56 | 0.545 |
| Male sex | 0.69 | 0.43–1.11 | 0.127 |
| Malignancy type (hematologic vs. solid) | 1.58 | 0.87–3.04 | 0.174 |
| CCI (unadjusted for age) > 4 | 1.04 | 0.57–1.92 | 0.894 |
| Troponin-I > 0.05 ng/mL | 2.05 | 1.06–3.97 | 0.034 |
