# Supplementary material for: Cardiac Troponin Is a Predictor of Septic Shock Mortality in Cancer Patients in an Emergency Department: A Retrospective Cohort Study
Source: PLoS One. 2016 Apr 14;11(4):e0153492. doi: 10.1371/journal.pone.0153492 (PMC4831781; doi:10.1371/journal.pone.0153492)
Supplement: S3 Table — (DOCX) [file pone.0153492.s007.docx]

| **Patient characteristic** | **Odds ratio** | **95% CI** | ***P* value** |
| --- | --- | --- | --- |
| PIRO2011 | 1.23 | 1.17–1.3 | < 0.001 |
| Age > 65 years | 1.01 | 0.99–1.0 | 0.145 |
| Black race | 0.95 | 0.49–1.8 | 0.866 |
| Male sex | 0.69 | 0.43–1.1 | 0.124 |
| Malignancy type (hematological vs. solid) | 1.51 | 0.80–2.8 | 0.202 |
| CCI (unadjusted for age) > 4 | 1.02 | 0.55–1.9 | 0.941 |
| CK-MB > 6.3 ng/mL | 1.61 | 0.99–2.6 | 0.055 |
