# Supplementary material for: Cardiac Troponin Is a Predictor of Septic Shock Mortality in Cancer Patients in an Emergency Department: A Retrospective Cohort Study
Source: PLoS One. 2016 Apr 14;11(4):e0153492. doi: 10.1371/journal.pone.0153492 (PMC4831781; doi:10.1371/journal.pone.0153492)
Supplement: S4 Table — (DOCX) [file pone.0153492.s008.docx]

| **Patient characteristic** | **Odds ratio** | **95% CI** | ***P* value** |
| --- | --- | --- | --- |
| MEDS | 1.31 | 1.22–1.42 | < 0.001 |
| Age > 65 years | 0.99 | 0.97–1.01 | 0.210 |
| Black race | 0.96 | 0.51–1.81 | 0.900 |
| Male sex | 0.88 | 0.55–1.42 | 0.610 |
| Malignancy type (hematologic vs. solid) | 0.61 | 0.33–1.10 | 0.100 |
| CCI (unadjusted for age) > 4 | 1.25 | 0.70–2.23 | 0.460 |
| Troponin-I > 0.05 ng/mL | 3.87 | 2.01–7.46 | < 0.001 |
