# Supplementary material for: Cardiac Troponin Is a Predictor of Septic Shock Mortality in Cancer Patients in an Emergency Department: A Retrospective Cohort Study
Source: PLoS One. 2016 Apr 14;11(4):e0153492. doi: 10.1371/journal.pone.0153492 (PMC4831781; doi:10.1371/journal.pone.0153492)
Supplement: S5 Table — (DOCX) [file pone.0153492.s009.docx]

| **Patient characteristic** | **Odds ratio** | **95% CI** | ***P* value** |
| --- | --- | --- | --- |
| MEDS | 1.28 | 1.20–1.37 | < 0.001 |
| Age > 65 years | 0.99 | 0.98–1.01 | 0.554 |
| Black race | 1.18 | 0.63–2.22 | 0.605 |
| Male sex | 0.89 | 0.55–1.43 | 0.637 |
| Malignancy type (hematologic vs. solid) | 0.75 | 0.41–1.38 | 0.353 |
| CCI unadjusted for age > 4 | 1.16 | 0.64–2.11 | 0.626 |
| CK-MB > 6.3 ng/mL | 2.21 | 1.37–3.57 | 0.001 |
